# A biopsychosocial approach assessing pain indicators among Black men

**DOI:** 10.3389/fpain.2023.1060960

**Published:** 2023-02-13

**Authors:** Tamara A. Baker, Knashawn H. Morales, Amber K. Brooks, Jaylyn F. Clark, Anna Wakita, Melicia C. Whitt-Glover, Yelia Z. Yu, Marcus Murray, Steven P. Hooker

**Affiliations:** ^1^ Department of Psychiatry, University of North Carolina, School of Medicine, Chapel Hill, NC, United States; ^2^ Department of Biostatistics, Epidemiology, and Informatics, University of Pennsylvania, Perelman School of Medicine, Philadelphia, PA, United States; ^3^ Department of Anesthesiology, Wake Forest University School of Medicine, Winston salem, NC, United States; ^4^ Wellstar Health System, Marietta, GA, United States; ^5^ University of North Carolina, Gillings School of Global Public Health Chapel Hill, Chapel Hill, NC, United States; ^6^ Gramercy Research Group, Winston-Salem, NC, United States; ^7^ Project Brotherhood, Chicago, IL, United States; ^8^ College of Health and Human Services, San Diego State University, San diego, CA, United States

**Keywords:** pain, black men, somatization, health, anxiety, depression

## Abstract

**Introduction:**

The lack of empirical evidence documenting the pain experience of Black men may be the result of social messaging that men are to project strength and avoid any expression of emotion or vulnerability. This avoidant behavior however, often comes too late when illnesses/symptoms are more aggressive and/or diagnosed at a later stage. This highlights two key issues - the willingness to acknowledge pain and wanting to seek medical attention when experiencing pain.

**Methods:**

To better understand the pain experience in diverse raced and gendered groups, this secondary data analysis aimed to determine the influence identified physical, psychosocial, and behavioral health indicators have in reporting pain among Black men. Data were taken from a baseline sample of 321 Black men, >40 years old, who participated in the randomized, controlled Active & Healthy Brotherhood (AHB) project. Statistical models were calculated to determine which indicators (somatization, depression, anxiety, demographics, medical illnesses) were associated with pain reports.

**Results:**

Results showed that 22% of the men reported pain for more than 30 days, with more than half of the sample being married (54%), employed (53%), and earning an income above the federal poverty level (76%). Multivariate analyses showed that those reporting pain were more likely to be unemployed, earn less income, and reported more medical conditions and somatization tendencies (OR=3.28, 95% CI (1.33, 8.06) compared to those who did not report pain.

**Discussion:**

Findings from this study indicate that efforts are needed to identify the unique pain experiences of Black men, while recognizing its impact on their identities as a man, a person of color, and someone living with pain. This allows for more comprehensive assessments, treatment plans, and prevention approaches that may have beneficial impacts throughout the life course.

## Introduction

There's an increasing body of literature showing that men are not only diagnosed with more terminal chronic diseases (e.g., heart disease, chronic obstructive pulmonary disease, cancer, HIV/AIDS) (1–3), but are more likely to experience greater pain intensity related to their medical diagnosis(es) (4, 5). While there are more scholarly data documenting disease-specific outcomes among men in general, discussing the pain experience of Black men remains understudied and inadequately assessed. This has not gone unnoticed, as emergent scholarly work has begun to refocus priorities examining the unique and complex interactions of social and behavioral health determinants in pain experiences among this raced and gendered group (6–10).

While lifestyle differences, marked by variability in socioeconomic status, financial hardship, food insecurity, medication costs, and other psychosocial characteristics, for example, are known to have a direct influence in the pain experience, less is known regarding the intersection of race, gender, and pain particularly among men from diverse race and ethnic groups (11–15).

The historical and social context of these identities (e.g., age, sex, abilities) have shaped how society perceives pain and health outcomes among those from racially minoritized groups. For example, during the time of slavery in the US and decades thereafter, there has been a long- held assumption that Black men and women are less likely to feel pain and to have a higher tolerance for pain than White men and women (16, 17). This is similarly generalizable to racially minoritized men, who are more likely to experience greater functional impairment due to their pain-related diagnosis, have limited access to resources, and experience significant barriers to pain care (18–20). While emergent data have focused predominately on behavioral and mental health (e.g., depression, anxiety) outcomes related to pain (21–25), more is now being done to understand the relationship between pain and social factors (e.g., perceived discrimination) among Black men (26). Although promising, what is known and understood about Black men and their experiences with pain remains scarce and is far below the margin to that of men who are not of the Black diaspora.

Yet, understanding the intersection of social, cultural, and behavioral domains as they influence the unique pain experiences of Black men is complicated and remains underdeveloped. Several reasons have been proposed as to why limited evidence of the pain experiences among Black men exists. First, men are often socialized to project strength, individuality, and avoid any expression of emotion or vulnerability, which could be interpreted as a weakness (27, 28). Second, while some may consider the masculine identity as a protective factor, the opposite may hold true, which often increases health risks, risk-taking behaviors, and emotional distress while diminishing health promoting behaviors (29). Third, since childhood many boys often mimic how to experience pain. Myers et al. (30), showed that boys who deviate from the socially “masculine role” (e.g., masking emotions, not crying) are often teased and/or rejected by their peers when physical pain is outwardly expressed. Additional pioneering work showed that hiding the emotional and physical expression and experience of pain validates behaviors whereby boys neither fear getting hurt, nor acknowledge their pain (31). As boys mature to adolescents, young men, and older adults, messages that accepting physical pain as a gesture of strength and resilience may prove harmful to their physical and mental health, social adjustment, and emotional well-being.

Given the potentially negative impact of ignoring pain, there remains the priority to understanding the pain experience of men in general, but more importantly the unique experiences of Black men. This, however, cannot be done if Black men are excluded from pain- related research and clinical trials. To address this need, the current study aimed to examine the influence identified demographic, somatization, and social and physical health indicators have in reporting pain among Black men 40+ years of age. Assessing the pain experience exclusively among Black men is a strength of this study and a contribution to the scientific literature.

## Methods

### Parent project

The current study includes a secondary analysis of baseline data from the parent project, the Active & Healthy Brotherhood (AHB) Study, a community-based randomized controlled trial of a 6-month culturally tailored intervention in Black men. Specific aims included testing: the immediate effects of the AHB intervention compared with the control condition on lifestyle behaviors and health-related outcomes, the immediate effects of the AHB intervention compared with the control condition on mediators of behavior change, and the longer-term (12 months post randomization) effects of the AHB intervention compared with the control conditions of all outcomes of interest.

Participants were recruited at in-person settings (e.g., community events, barber shops, churches, sporting activities, gas stations, fast food restaurants, car washes) from four cities in the southern part of the United States. Interested participants provided study recruiters with contact information that included name, telephone number and email address, best time to be contacted, and county of residence. Participants were also recruited *via* print, television, radio, newspaper, and social media advertisements. Advertising materials included a phone number to call a research team member for additional information about the study. Additional details of the larger parent study are described elsewhere (32).

### Current study

The current study was a secondary analysis of a limited selection of existing variables collected during the parent study, as described below.

### Pain outcome

Pain was assessed with a single-item question taken from the CDC Health-Related Quality of Life Questionnaire (33); In the past 30 days, have you had any physical pain or health problems that made it hard for you to do your usual activities such as driving, working around the house, or going to work?. Response choices were dichotomized as “no/not sure/unknown” and “yes”.

### Social and behavioral indicators

Social and behavioral indicators included depression, anxiety, and somatization subscales of the Brief Symptom Inventory (BSI) and the Perceived Stress Scale (PSS). The Brief Symptom Inventory (BSI) is a 53-item short form of the Symptom Checklist 90-Revised and BSI (34). Each item was scored on a 5-point Likert scale of distress, with coefficients ranging from.79 to.85 on the depression scale. Stress was assessed *via* the Perceived Stress Scale (PSS), a 14-item self- report scale measuring how different situations affect one's feelings and perceived stress. Scores ranged from 0 to 40, with higher scores endorsing greater perceived stress (35).

The Holmes-Rahe Stress Inventory was included to measure “life events” that happened to participants during the previous year, with each situation having a designated point value (36). Examples of these events included, but are not limited to, marriage, death of spouse, and changes in residence. The final score provided an estimated outlook for the participant to have a “stress- induced health breakdown”.

### Demographic and health variables

Analyses included several covariates and potential confounding variables: marital status, education, income below the national poverty line according to household size, and employment status (37). Marital status was assessed as never married, married, separated, divorced/widowed/unmarried partner/other. Education was categorized as high school graduate or less and Bachelor's degree or more. Family income was dichotomized as yes/no in living below the national poverty line. Employment status was assessed as yes/no in being currently employed.

The CDC Health-Related Quality of Life measure assessed self-reported health status.

On a five-point Likert scale, response choices included: excellent, very good, good, fair, or poor. The total number of pain-related medical conditions (arthritis, high blood pressure, diabetes, obesity, angina, cancer) was included in the final statistical model.

### Statistical analyses

Bivariable analyses were calculated including demographics, general health, and psychosocial measures comparing pain groups (with and without) using the Wilcoxon rank-sum test or an exact test as appropriate for continuous and categorical factors. A multivariable logistic regression model was used to examine the associations between psychosocial measures and pain while adjusting for potential confounders. Results are presented as odds ratios and 95% confidence intervals where the statistical significance level was set at 0.05. Final analyses were limited to participants with complete data on the measures of interest.

## Results

### Current study

Of the 333 men randomized for the parent AHB project, 12 (4%) were excluded from the current analyses due to missing data. For subsequent analyses (*n* = 321), more than half of the men were married (54%) and employed (53%), with an average age of 50.73. The majority of the Black men in this sample earned income above the federal poverty level (76%), and self-rated their overall health as good/better (75%). Less than one-third of the men (22%) men reported pain in the last 30 days.

Education, employment status, income, self-rated quality of life, and number of medical conditions differed by pain groups (pain vs. no pain) and were included in the multivariable model. Data further showed that marital status did not differ between the two groups, with those who reported pain being less likely to be married (47% vs. 55%, *p* = .37). Descriptive analyses also showed that men reporting pain completed less years of education compared to the no pain group (26% vs. 18%, *p* < .05). Similarly, men with no pain reported better health (29% vs. 15%; *P* < .05), and were more likely to be employed than those reporting pain (69% vs. 41%, <.001).

Additionally, a significant difference was found in medical illnesses, with pain being associated with an arthritis diagnosis (43% vs. 14%; < .001;). Additional demographic data are provided in Table 1.

**Table 1:** Sociodemographic and health characteristics (*n* = 321).

		Total sample (*n* = 321)	Pain in the last 30 days	
			No (*n* = 249)	Yes (*n* = 72)
**Demographic characteristics**				
Marital status	Never married	66 (20.6%)	50 (20.1%)	16 (22.2%)
	Married	172 (53.6%)	138 (55.4%)	34 (47.2%)
	Separated	19 (5.9%)	16 (6.4%)	3 (4.2%)
	Divorced/widowed/unmarried partner/other	64 (19.9%)	45 (18.1%)	19 (26.4%)
Education	High school graduate or less	64 (19.9%)	43 (17.3%)	21 (29.2%)
	Bachelor's degree or more	135 (42.1%)	113 (45.4%)	22 (30.6%)
Employment status	No	152 (47.4%)	102 (41.0%)	50 (69.4%)
	Yes	169 (52.6%)	147 (59.0%)	22 (30.6%)
Household size, median (IQR)		2.0 (1.0, 3.0)	2.0 (1.0, 3.0)	2.0 (1.0, 3.0)
Below poverty line (2022)	No	245 (76.3%)	198 (79.5%)	47 (65.3%)
	Yes	76 (23.7%)	51 (20.5%)	25 (34.7%)
**Health and quality of life indicators**				
Self-rated QOL	Excellent/very good	85 (26.5%)	74 (29.7%)	11 (15.3%)
	Good	156 (48.6%)	115 (46.2%)	41 (56.9%)
		Total sample (*n* = 321)	Pain in the last 30 days
	Fair/poor	80 (24.9%)	60 (24.1%)	20 (27.8%)
# Medical Conditions (max 5), median (IQR)		1.0 (0.0, 2.0)	1.0 (0.0, 2.0)	2.0 (1.0, 3.0)
Arthritis	No	245 (78.5%)	205 (85.1%)	40 (56.3%)
	Yes	67 (21.5%)	36 (14.9%)	31 (43.7%)
High Blood Pressure	No	165 (51.7%)	135 (54.2%)	30 (42.9%)
	Yes	154 (48.3%)	114 (45.8%)	40 (57.1%)
Diabetes	No	253 (79.8%)	201 (81.7%)	52 (73.2%)
	Yes	64 (20.2%)	45 (18.3%)	19 (26.8%)
Obesity	No	206 (64.8%)	166 (67.2%)	40 (56.3%)
	Yes	112 (35.2%)	81 (32.8%)	31 (43.7%)
Angina	No	301 (94.7%)	235 (94.8%)	66 (94.3%)
	Yes	17 (5.3%)	13 (5.2%)	4 (5.7%)
Cancer	No	304 (95.0%)	239 (96.0%)	65 (91.5%)
	Yes	16 (5.0%)	10 (4.0%)	6 (8.5%)
**Behavioral and psychosocial indicators**				
Holmes_Rahe_Stress_Scale, median (IQR)		170.0 (94.0, 288.0)	166.0 (92.0, 288.0)	186.5 (101.5, 291.0)
		Total sample (*n* = 321)	Pain in the last 30 days
Holmes_Rahe_Stress_Scale_Level	High	77 (24.0%)	60 (24.1%)	17 (23.6%)
	Moderate	101 (31.5%)	76 (30.5%)	25 (34.7%)
	Low	143 (44.5%)	113 (45.4%)	30 (41.7%)
Depression, median (IQR)		0.0 (0.0, 0.3)	0.0 (0.0, 0.3)	0.2 (0.0, 0.7)
Anxiety, median (IQR)		0.2 (0.0, 0.3)	0.2 (0.0, 0.3)	0.2 (0.0, 0.5)
Somatization, median (IQR)		0.1 (0.0, 0.4)	0.1 (0.0, 0.4)	0.4 (0.1, 0.8)
Perceived_Stress_Score, median (IQR)		19.0 (12.0, 24.0)	18.0 (12.0, 24.0)	20.5 (14.0, 25.5)
Global severity index, median (IQR)		10.0 (4.0, 21.0)	8.0 (3.0, 20.0)	15.5 (7.5, 28.5)
PST, median (IQR)		10.0 (4.0, 19.0)	8.0 (3.0, 19.0)	15.5 (8.0, 23.0)
PSDI, median (IQR)		1.0 (0.9, 1.2)	1.0 (0.9, 1.2)	1.0 (0.9, 1.3)

Table 2 shows that after adjusting for confounding factors (demographic, education, health, income) and other sociodemographic variables, Black men with higher somatization scores were more likely to self-report pain in the past 30 days compared those not reporting pain (OR = 3.28, 95% CI (1.33, 8.06). No statistical evidence was found documenting an association between depression, anxiety, or stress with pain.

**Table 2:** Association between pain and psychosocial factors adjusted for potential confounders.

Variables	Odds ratio	95% Confidence Interval		*p*-value
Depression	1.46	0.65	3.27	0.36
Anxiety	0.85	0.27	2.65	0.77
Somatization	3.28	1.33	8.06	0.01
Stress	0.99	0.95	1.04	0.69
**Marital Status**				
Never married	Reference	0.73
Married	0.89		0.37	2.14
Separated	0.47		0.10	2.29
Divorced/widowed/unmarried partner/other	1.12		0.46	2.76
**Education**				
High school graduate or less	Reference	0.83
Associate's Degree or some college (no deg)	0.84		0.38	1.84
Bachelor's degree or more	0.73		0.31	1.71
Other	1.48		0.20	11.01
**Employed**				
No	Reference	0.02
Yes	0.48		0.25	0.91
**Income below poverty level**				
No	Reference	0.55
Yes	1.30		0.56	3.03
Number of medical conditions	1.31	1.01	1.68	0.04
**Quality of Life**				
Excellent/very good	Reference	0.06
Good	2.11		0.96	4.64
Fair/poor	1.06		0.42	2.71
**The Holmes and Rahe Stress Scale Level**				
High	Reference	0.87
Moderate	1.19		0.54	2.64
Low	1.22		0.56	2.66

*Multivariable logistic regression model was adjusted for marital status, education, employment status, income below the poverty level, number of medical condition, quality of life, and life events.

## Discussion

Traditional pain models have yet to capture the pain experiences of Black men. This may be due to the absence of representation in pain studies, the lack of interest in the health and social well-being of Black men, and/or adhering to false narratives that men and Black individuals do not experience pain (or of less intensity that other race groups). These assumptions remind us that collectively, the unique experiences of pain among Black men have been largely ignored.

Results from the current study showed a relationship between reported pain and chronic illnesses and other life events. Because of the limited data documenting the pain experience of Black men, we cannot show if our data are consistent with other studies assessing pain specifically among this gendered group. We can however, report that findings from this investigation are consistent with comparative studies showing differences in health outcomes between men and women. Data show that although men are less likely to present with moderate to severe pain interference (i.e., disturbances to daily activities, life roles, moderate to severe employment, and interpersonal relationships) (38), they also endorse delayed pain thresholds (i.e., the point at which one first detects pain) compared to women. Emergent data concurs with these observations documenting the role social and demographic factors have in the daily lived experiences of those reporting pain. (Blinder's (2022)) study, for example documented a significant association between pain, employment, and financial status (39). Others have found similar relationships across socioeconomic groups, gender/sex, and race groups (40–43).

Similar findings from our study exhibit differences in employment and income between the two pain groups. These differences may not only be explained by one's job positioning, but also by the job's responsibilities and day-to-day tasks. This suggests that those experiencing pain may not be able to continue performing and/or keep up with the (mental/physical) demands of their current employment. As a result, these individuals may take on less hours which can impact their earned salary. Similarly, if an individual is unable to keep up with the demands of a job, particularly if the job requires physical exertion, they may opt to quit/resign or are fired.

The outcome resulting from the physical demands of the job, may also be related to the medical diagnosis of some of the Black men. Our results also showed that those who reported pain were more likely to self-report having arthritis. This is consistent with the abundance of data documenting the significant relationship between with osteoarthritis, rheumatoid arthritis, and other arthritis-related diseases and pain (44, 45). More importantly, it documents the outcome of this debilitating medical diagnosis and the impact it may have on employment, job satisfaction, income, and wealth.

Another interesting finding from the current study is that those reporting pain had higher somatization (i.e., reporting a medical symptom with no physiologic evidence) scores. This is consistent with other studies documenting the significance between pain and somatization, and other determinants (e.g., depression, anxiety) in clinical and community settings (46, 47). While a subjective experience, the tendency to report pain without any physiological evidence may result in being mis-diagnosed and/or have their pain under (over) treated and not appropriately managed. Yet, while some consider somatization as an exaggeration of health complaints, others may interpret these actions as a means of coping with their medical symptoms. Drawing attention to how one is feeling and/or experiencing certain symptoms may result in being believed that their pain exists. This may result in their pain being properly treated and managed. While speculative, this important finding (and explanation) is key in addressing how Black men may interpret their health and well-being. Including somatization in these analyses may lead to other discussions on the impact behavioral and psychologic influences have in assessing pain.

For example, we need to start framing our discussions around the relationship(s) between emotional trauma, somatization, and physical pain among Black men. This may provide for a better understanding of their health and help seeking behaviors regarding their pain.

Pain is a dynamic illness and/or symptom that is neither linear nor singular in occurrence.

Understanding the complexity of addressing these outcomes, particularly among groups with multiple intersecting identities (e.g., male/man, Black, older, etc.) requires an understanding of experiences that are unique to that particular group. This requires scholars to move beyond the traditional comparative methodologies when conducting research among racially and/or gendered minoritized groups. Results from this study remind us that it is critical to understand *how* and *why* Black men experience pain (48). This cannot be done if the research follows a deficit model approach, whereby if comparing the pain experiences of one race group to that of another race group, the result is that one group does not do as well compared to the other. This is often done when assessing health outcomes by race group. (Baker and Gamaldo (2022)) highlight strategies and benefits of conducting statistically appropriate within race group research without having to compare with another group (49). This work presents information on the most effective methodological approach to conducting race-based research.

This approach reminds us that pain is not a one-size-fits- all, but rather a grouping of factors the influence when pain is reported, how it's experienced, why it's reported, to whom it's reported to, and where and which type of services are sought in having pain diagnosed, treated, and managed. Specific to Black men, data are urgently needed to clarify intra-group (within) differences to explore how lived experiences (and changes in lived experiences) differ between Black men who live with pain and those who don't, and to consider how these differences can be leveraged to improve pain care.

It is without question that significant gaps remain regarding adequate conceptualization of psychosocial variables that contribute to pain inequities (50). Disparities and inequities in pain care are created and maintained by intersecting levels of social structures; therefore, true equity is more likely to be addressed through efforts focused on exploring and addressing psychosocial influences of inequity This expands on current pain models, which may expose themes related to the psychosocial experiences of Black men living with pain has the potential to elucidate specific mechanisms that can be leveraged to deliver care that is more effective and equitable.

### Strengths

Findings from this study are an appealing addition to the scientific literature, given that this is one of very few projects that focus on the pain experience exclusively among Black men. This is significant considering that little effort has attended to this cohort of men. There are several reasons that may attribute to this (un)intentional outcome: (a) may not be of interest to the primary investigator/research team, (b) claims that Black men are difficult to recruit, (c) little effort to retaining Black men for projects, and (d) not able to recruit enough Black men for adequate representation. Our efforts to focus on the pain needs of Black men questions why so little attention has been given to this gendered and raced group. Scholars such as Drs. Wizdom Powell, Derek Griffith, Roland Thorpe, and Daphne Watkins, to name a few, continue to galvanize the field with their novel research focusing on health outcomes among Black men.

Yet, few have focused exclusively on the unique pain experiences of these men, hence the significance of the current project.

### Limitations

While these data provide a significant contribution to the pain literature, such as being one of the first to focus on the unique pain experience of middle-aged and older Black men, the limitations of the study should be identified. First, analyses included a single-item pain question, therefore pain severity and intensity could not be assessed. It's important that future analyses include multiple questions/measures to capture a more coherent and comprehensive assessment of the pain experience. Second, data were self-reported which may result in potential reporting bias such as social desirability. Third, there should be some caution in interpreting results of this study and/or generalizing the findings to other race, age, or gender/sex groups. Also, this sample is from the US, and therefore there is limited generalizability regarding these results in more collectivistic cultures; in particular, those with state-funded, or more readily accessible, health services. Another limitation is that we neither assessed the type of self-reported arthritis nor provided information on the treatment and health care utilization of the men in our sample This is important given that the type can impact affected joints, severity, frequency, and intensity.

This may also influence other social factors such as income and employment. It's important that future investigations assess the type and for how long the individual been diagnosed with the disease. Finally, while of significance, another limitation is that somatization has not been extensively examined in Black men. Therefore, we must be careful in how these findings are interpreted, particularly as it relates to pain outcomes.

## Conclusion

Results from the current study showed that Black men reporting pain were more likely to report a higher prevalence of somatization, and were more likely to be unemployed, have less income, and self-report being diagnosed with arthritis. With these findings, efforts are needed to chronicle what it means for Black men to (1) interpret and experience (chronic) pain, (2) understand the impact pain has in their identity as a Black man, (3) establish best practices that conform to more adaptive and healthy coping skills, and (4) identify constructs that influence their daily lived experiences with (multiple) chronic pain illnesses. This builds on (Keogh's (2015)) scholarly work that questions the “next steps” in addressing the needs of (Black) men experiencing chronic pain: what are the health needs and policy implications for the management of men's pain?; should there be specific interventions [with a personalized treatment approach] for chronic pain?”; and can a men's health approach be used to help improve treatment?” (51). These questions acknowledge the relationship between pain and masculine expression, thereby recognizing pain as dynamic and multidimensional.

Answering these questions requires a set of immediate-, short-, and long-term goals that can be applied in both clinical and community settings. This suggests that research should not only focus on the “masculine” expression of pain, but “how” pain is experienced. In assessing the “how”, it's important that we direct our attention to identify which (if any) factors influence the pain experience. We can no longer apply a universal approach to how Black men's pain is diagnosed, treated, and managed. Yet, to move beyond this more traditional approach, Black men must be equally represented in pain research, clinical trials, and/or are aware of specialty pain care/treatment.

Addressing these outcomes requires a change in how we recognize and accept the pain experience of men, while also endorsing messages that experiencing pain is “normal” and a part of human development. By dismissing the stereotypes associated with masculinity and health outcomes, we can comprehensively address the meaning of pain while acknowledging the need for more rigorous research and preventative and rehabilitative care models for pain care.

## Data Availability

The original contributions presented in the study are included in the article/**Supplementary Materials**, further inquiries can be directed to the corresponding author/s.
