# Supplementary figures and images for: A biopsychosocial approach assessing pain indicators among Black men
Source: Front Pain Res (Lausanne). 2023 Feb 13;4:1060960. doi: 10.3389/fpain.2023.1060960 (PMC9968840; doi:10.3389/fpain.2023.1060960)

## CONSORT Flow Diagram

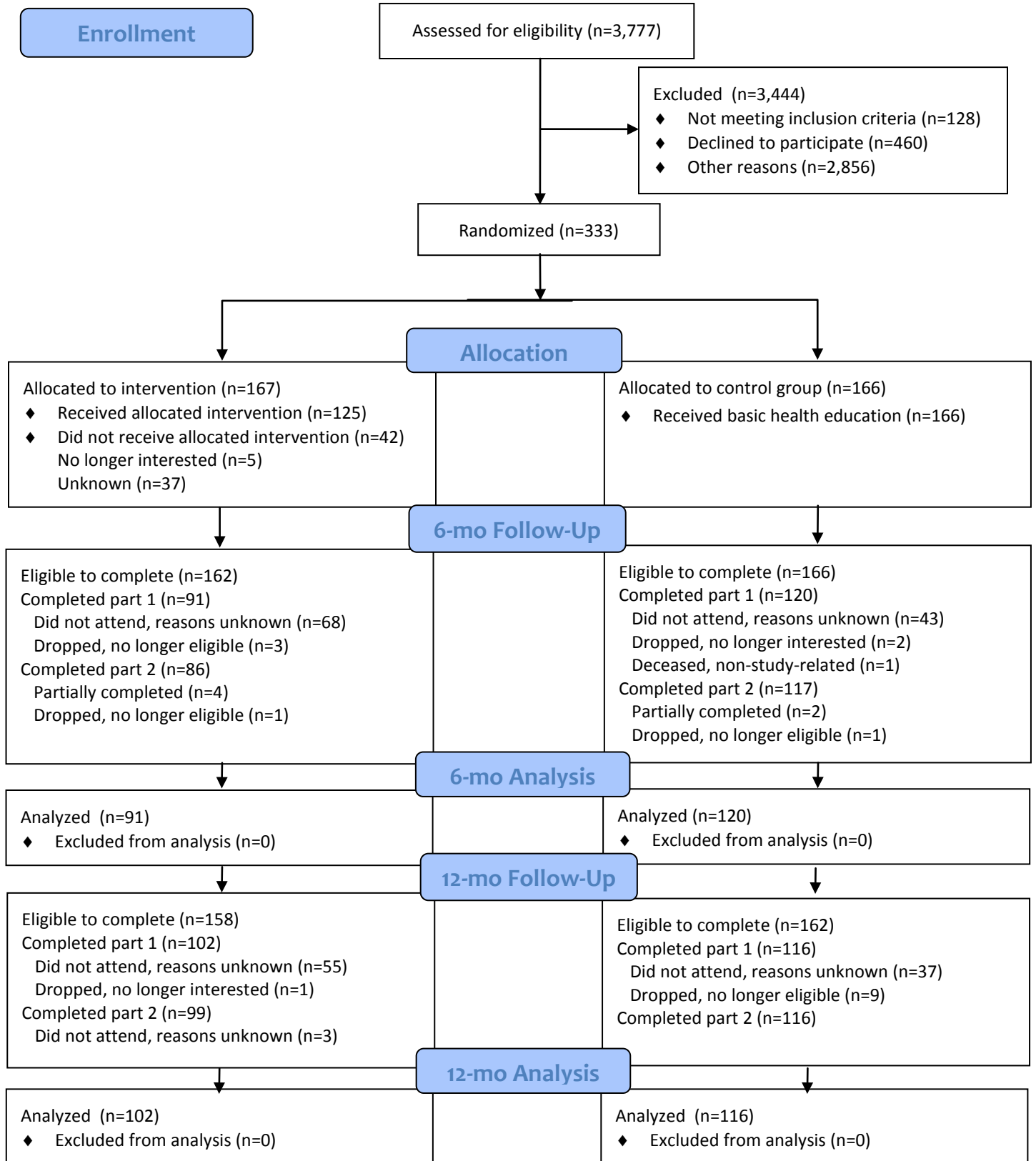

Supplement: Supplementary file 1 [file Datasheet1.pdf]
